# Socioeconomic disparities in basic life support awareness and training among Saudi adults: a cross-sectional study

**DOI:** 10.7717/peerj.20678

**Published:** 2026-01-27

**Authors:** Mohammad A. Jareebi, Mohammed H. Ghasham, Naif M. Alshamrani, Turki I. Aljezani, Amani A. Mutaen, Yara A. Mutaen, Faris A. Alhazmi, Ryof M. Sahli, Ahmed Y. Alkadi, Majed A. Ryani, Ahmed A. Bahri, Ahmad Y. Alqassim, Nuha H. Abutalib, Mostafa Mohrag, Abdulrahman S. Hamdi

**Affiliations:** 1 Faculty of Medicine, Jazan University, Jazan, Saudi Arabia; 2 Department of Family and Community Medicine, Faculty of Medicine, Jazan University, Jazan, Saudi Arabia; 3 Department of Medicine, Faculty of Medicine, Jazan University, Jazan, Saudi Arabia

**Keywords:** Basic life support (BLS), Cardiac arrest, Public awareness, CPR training, Emergency response, Cross-sectional study, Knowledge assesment, Blood circulation, Automated external defibrillators (AEDs), Chest compressions

## Abstract

**Background:**

Basic life support (BLS) is a critical emergency intervention that significantly enhances survival rates in situations such as cardiac arrest. Bystander cardiopulmonary resuscitation (BCPR) enhances the chances of survival up to 24%. This study aimed to assess public awareness, knowledge, and attitudes toward BLS, as well as the factors influencing them in the Jazan region of Saudi Arabia.

**Methods:**

An analytical cross-sectional study was conducted in the Jazan region during October 2024 and March 2025. A standardized questionnaire was used to evaluate participants’ BLS knowledge. Eligible participants included mentally competent Saudi and non-Saudi residents aged 18 years and older. Descriptive statistics and multiple linear regression analyses were performed, with a significance level set at *p* < 0.05.

**Findings:**

A total of 1,021 participants were included, with a mean age of 30 ± 11 years; 51% were female. Overall, 72% reported awareness of BLS, and 56% had received prior BLS training. However, only 5% had engaged in hands-on practice. Educational institutions (34%) and social media platforms (20%) were the most frequently cited sources of BLS knowledge. The majority of the participants were aware of cardiac arrest symptoms (80%), but more than half lacked practical knowledge of BLS (>50%). Higher BLS knowledge scores were significantly associated with holding a bachelor’s degree (β = 0.68; *p* < 0.001), higher income levels (β = 2.48; *p* < 0.001), and engaging in moderate to vigorous physical activity at least five times per week (β = 0.82; *p* = 0.019). Conversely, having children was negatively associated with BLS knowledge (β = −2.31; *p* = 0.002).

**Conclusion:**

Socioeconomic factors such as income, education, smoking status, academic background, physical activity and having children showed a significant associated with BLS knowledge, highlighting the need for broader public education and accessible BLS training programs.

## Introduction

Cardiac arrest, defined as sudden cessation of effective blood circulation, remains one of the leading causes of death globally. Out-of-hospital cardiac arrest (OHCA), in particular, continues to show critically low survival rates. In the United States, an estimated 356,000 OHCA cases occur annually, with survival rates ranging from only 2% to 11%. The 2023 heart disease and stroke statistics state that among the over 356,000 OHCA that occurred, 40.2% received bystander cardiopulmonary resuscitation (CPR) (Mehra, 2007; Berdowski et al., 2010; American Heart Association, 2021).

Basic life support (BLS) is the immediate care given during an emergency or injury, typically delivered by a bystander prior to the initiation of professional medical assistance. Immediate bystander intervention is essential in improving survival outcomes. Therefore, sufficient knowledge of BLS is essential for individuals to effectively provide life-saving interventions during emergencies (Shaheen et al., 2023). BLS measures—such as cardiopulmonary resuscitation (CPR) and the use of automated external defibrillators (AEDs)—have been proven to double or even triple the chances of survival. Bystander CPR was associated with a 24% higher likelihood of surviving to hospital discharge (Chan et al., 2023). However, there is a stark global disparity in BLS knowledge and practical application, highlighting widespread systemic gaps in public preparedness (Ministry of Health, Saudi Arabia).

BLS involves vital interventions including chest compressions, rescue breathing, airway management, and defibrillation, all crucial for maintaining oxygen delivery during cardiac emergencies (Koster, 2013). Despite its lifesaving potential, public competency remains alarmingly low. According to a survey conducted on general population of Turkey, only 3.6% of individuals had performed CPR in an emergency, and fewer than 40% had received CPR training (Ozbilgin et al., 2015). Similar deficits have been reported in Spain, 43% of adults surveyed were unfamiliar with basic first aid protocols (García-Blaya, Abraldes & Vaquero-Cristóbal, 2025). These findings emphasize the urgent need for standardized, accessible BLS training programs worldwide.

Previous studies have demonstrated that BLS knowledge and performance outcomes are primarily influenced by educational background, training, parental status and prior exposure. In Singapore, 55% of parents passed the basic infant BLS assessments. Higher educational attainment was a significant factor impacting all scores (Chia & Lian, 2014). Physical fitness and regular physical activity have been shown to influence the quality of BLS performance. In particular, studies assessing CPR skills have found that participants with higher physical activity levels achieved a significantly greater percentage of chest compressions with adequate depth compared to those with lower activity levels (97% *vs.* 87.8%, *P* = 0.003) (Ippolito et al., 2021).

According to the Saudi OCHA registry, the majority of OHCAs (82.6%) occur at home, with a reported mortality rate of 13.3% (Alabdali et al., 2024). In addition to knowledge deficits, cultural and psychological barriers—such as fear of legal consequences, causing harm, or personal discomfort—discourage many from performing CPR. Gender disparities also influence bystander CPR (BCPR) rates. Cultural beliefs, practices, and laws disproportionately affect females and they are significantly less likely to receive BCPR during OHCA incidents than males (31.2% *vs* 36.4%) (Liu et al., 2022). This discrepancy has been linked to social and cultural factors, including perceived physical fragility of women, concerns about chest exposure, gender stereotypes and pregnancy condition. Concerns about sexualization of female’s bodies and the false belief that women are less likely to experience cardiac arrest further contribute to the issue (Chen et al., 2024). These challenges are compounded by unequal access to AEDs and the absence of regular refresher training, suggesting that current educational strategies fail to ensure skill retention and real-world readiness (Alghamdi et al., 2024; Almutairi et al., 2023; Aldhakhri, 2020).

In this context, the Jazan region presents a particularly critical case. It is a socioeconomically diverse area with unique cultural and geographic dynamics. Studies indicate that 68% of residents live in areas with limited access to healthcare services, pointing to potential deficiencies in emergency response infrastructure. Moreover, the region exhibits a high prevalence of cardiovascular risk factors, with hypercholesterolemia affecting 53% of adults, indicating a heightened risk for cardiac events (Ghazwani et al., 2023). While national data highlight general shortcomings in BLS awareness, localized information specific to Jazan is sparse. There is a lack of detailed insight into public perceptions, exposure to BLS training, and region-specific barriers that might hinder implementation and participation. The International Liaison Committee on Resuscitation (ILCOR) also identifies a gap in knowledge and training for bystanders to recognize cardiac arrest and act accordingly (Kleinman et al., 2018).

This study aims to address that gap by assessing public knowledge, attitudes, and practices regarding BLS in the Jazan region. The findings will help inform targeted community-level interventions to strengthen emergency preparedness. Ultimately, these efforts align with Saudi Vision 2030’s broader goals of healthcare modernization and community empowerment.

## Materials and Methods

### Study design and participants

This analytical cross-sectional and questionnaire-based study was carried out in Jazan, Saudi Arabia between October 2024 and March 2025. Data was obtained through an online, self-administered questionnaire distributed to individuals who voluntarily agreed to participate.

Eligible participants included mentally competent non-Saudi and Saudi nationals aged 18 years and above regardless of gender. Participation was entirely voluntary, and informed consent was obtained prior to inclusion. Individuals under the age of 18, those who declined participation, or those who were unable to complete the questionnaire were excluded from the study.

The required sample size was determined using the Cochran formula, based on a 95% Cl, a 5% margin of error, and an assumed response distribution of 50%. To compensate for an anticipated 25% non-response rate, the minimum final sample size was calculated to be 513 participants. A non-probability convenience sampling method was employed for participant recruitment. The sample size was calculated using the following formula:

\begin{eqnarray*}n= \frac{{Z}^{2}\cdot p\cdot (1-p)}{{E}^{2}} \end{eqnarray*}

where:

• *n* = required sample size
• *Z* = score corresponding to the desired confidence level (1.96 for 95% confidence)
• *p* = expected prevalence or response distribution (0.5, used for the most conservative estimate)
• *E* = margin of error (0.05)

### Data collection tool

A self-developed, self-administered questionnaire was created after an extensive review of the American Heart Association (AHA) requirements for BLS to collect data (American Heart Association, 2020). The instrument was designed in Arabic to ensure accessibility and comprehension among the target population. The questionnaire comprised three main sections: (1) sociodemographic and health characteristics, (2) BLS perception and practice, and (3) knowledge, attitudes, and awareness regarding BLS.

• **Sociodemographic and health characteristics:** This section included 15 items gathering data on participants’ age, gender, place of residence, weight, height, nationality, marital status, number of children, educational attainment, occupation, monthly income, smoking status, exercise habits, and personal or family history of chronic illnesses.
• **Knowledge, attitudes, and awareness regarding BLS:** This component included 21 items assessing knowledge of cardiac arrest signs and symptoms, prior CPR training, correct hand placement and compression rate, concerns about making mistakes during CPR, definition of cardiac massage, performance of respiration and cardiac massage, actions to take if a family member or friend faints, BLS applications in cardiac arrest, correct compression-to-ventilation ratio, appropriate area, rate, and force for cardiac massage, training and learning exposure, and familiarity with related devices and equipment.
• **BLS perception and practice:** This section consisted of nine items aimed at BLS awareness, knowledge source, importance of BLS, necessity of BLS, number of BLS trained individuals, training time, acquisition of mandatory or optional learning, training method and facing CPR situation.

BLS knowledge was quantified by assigning one point for each correct response and zero points for incorrect answers, yielding a total score range of 0 to 20. The sum of the correctly answered knowledge questions was normally distributed. Correct answers referred to respondents who correctly answered >50% of the total knowledge question (Tadesse et al., 2022).

To ensure the tool’s validity, reliability, and clarity, a pilot test was conducted among 25 participants. A survey was conducted on the small group first. This helped identify confusing questions, poor wording, or technical issues. Additionally, it reduced response bias. Based on their feedback, minor revisions were made to enhance the questionnaire before final deployment. Participants were recruited using a convenience sampling method. The online questionnaire was distributed through widely used social media platforms such as Telegram, WhatsApp, X (formerly Twitter), Facebook, and other widely used channels, which reduced sampling bias. Individuals who voluntarily agreed to participate accessed the survey link and submitted their responses. To minimize social desirability bias, no personal identifiers were collected. Participants were presented with a clear description of the goals of study and informed permission was gained before participation. Throughout the research process, strict measures were taken to maintain participant privacy and confidentiality (Jarrah, Judeh & AbuRuz, 2018).

The survey and its scoring system were piloted on a small sample to test feasibility. Researchers analyzed whether the scores worked as intended, differentiated between respondents, and identified ambiguous answers. The scoring criteria were then refined based on these findings. To ensure the correctness of the data collected through the online survey, clear and concise instructions were provided to participants at the beginning of the survey to minimize misinterpretation of the questions. Mandatory fields were included to avoid missing data, and the system was configured to prevent multiple submissions from the same participant, thereby reducing the risk of duplication. The anonymity of participants was maintained to encourage honest responses and minimize the risk of social desirability bias.

### Data analysis

Following data collection, the raw information and data were transferred to Microsoft Excel (Microsoft, Redmond, WA, USA). Preliminary cleaning was conducted by identifying and excluding missing values and inappropriate entries. About 5 percent participants showed missing information. As a result, there were no missing data in the final dataset. Determinants were selected based on evidence from existing literature and their theoretical relevance to BLS knowledge. Key demographic and demographic variables (age, gender, education, healthcare background, and prior BLS exposure) were included to account for potential confounding and to ensure a comprehensive assessment of factors influencing knowledge levels.

Statistical analyses were conducted employing R software (Version 4.2.3; R Foundation for Statistical Computing, Vienna, Austria). Descriptive statistics including percentages, standard deviations and averages were performed depending on the nature of each variable. To evaluate associations between the BLS knowledge score and potential determinants, multiple linear regression analysis was conducted. Multiple linear regression was chosen because the study aimed to assess the independent effect of several predictor variables (*e.g.*, education, income, physical activity, smoking status) on a single continuous outcome variable (BLS knowledge score). Statistical significance was set at *p* < 0.05 for all analyses.

### Ethical consideration

Ethical permission for the research was received from the Research Ethics Committee (REC) of Jazan University, before data collection (Reference No. REC-46/04/1203; dated 06/10/2024). All necessary institutional permissions were secured, and the study objectives were clearly communicated to all prospective participants. Participation was entirely voluntary, and individuals were assured of their anonymity, confidentiality, and freedom from any sort of harm. Written informed consent was obtained from all participants. The cross-sectional study was carried out in conformity with the ethical standards established in the Declaration of Helsinki and was presented following the Strengthening the Reporting of Observational Studies in Epidemiology (STROBE) guidelines to maintain methodological rigor and transparency.

## Results

### Sociodemographic characteristics

The current study included 1,021 individuals (mean age: 30 ± 11 years; 51% females), The participant pool predominantly consisted of Saudi nationals (*n* = 995, 97%) including urban as majority (*n* = 528; 52%). Of the total sample, 635 participants (62%) were unmarried. Analysis showed that the participants were predominantly bachelor’s degree holders (*n* = 663; 65%), and nearly half were students (*n* = 501; 49%). Regarding monthly income, 344 (34%) participants reported earning less than 5,000 SAR and 294 (29%) fell within the 5,000 to 15,000 SAR range (Table 1).

**Table 1:** Sociodemographic characteristics of the sample (*n* = 1,021).

Characteristics	**Mean ± SD**
**Age**	30 ± 11 years
**Characteristics**	**Frequency (%)**
**Sex**	
*Female*	518 (51%)
*Male*	503 (49%)
**Nationality**	
*Saudi*	995 (97%)
*Non-Saudi*	26 (3%)
**Residence**	
*Rural*	493 (48%)
*Urban*	528 (52%)
**Education**	
*Bachelor’s degree*	663 (65%)
*High school or less*	312 (31%)
*Postgraduate studies*	46 (5%)
**Employment**	
*Employee*	337 (33%)
*Student*	501 (49%)
*Unemployed*	183 (18%)
**Marital status**	
*Non-Married*	635 (62%)
*Married*	369 (36%)
*Divorced/widow*	17 (2%)
**Family income**	
*Less than 5,000*	344 (34%)
*5,000–9,999*	190 (19%)
*10,000–15,000*	193 (19%)
*>15,000*	294 (29%)
**Children**	
*No*	108 (11%)
*Yes*	343 (33%)

**Notes.**

Abbreviations SD Standard deviation n Sample size

Participants had a mean weight of 69 ± 20 kg and a mean height of 164 ± 11 cm, corresponding to an average BMI of 25.59 ± 6.7. Most (*n* = 82; 81%) were non-smokers, while approximately two-thirds reported having a family history of chronic diseases. Regarding physical activity, 442 (43%) reported being inactive. Additionally, 33% of participants reported participating in risky sports (Table 2).

**Table 2:** Health-related profile of the participants (*n* = 1,021).

Characteristics	**Mean ± SD**
*Weight*	69 ± 20 kg
*Height*	164 ± 11 cm
*BMI*	25.59 ± 6.7 kg/m^2^
**Characteristics**	**Frequency (%)**
**Smoking**	
*Never*	828 (81%)
*Current*	127 (12%)
*Ex-smoker*	66 (6%)
**Chronic diseases in the family**	
*No*	342 (33%)
*Yes*	679 (67%)
**Physical activity**	
*No physical activity*	442 (43%)
*Moderate to intense physical activity (*≤*30 min, 5x/ week)*	206 (20%)
*Moderate to intense physical activity (<30 min, 5x/ week)*	373 (37%)
**Risky sports**	
*No*	685 (67%)
*Yes*	336 (33%)

### Knowledge, attitudes, and awareness regarding BLS

The majority of participants (*n* = 817, 80%) were able to identify concerns that would prevent cardiac massage and could recognize the signs of sudden cardiac arrest. However, deficits were observed in the initial assessment of a victim. Nearly a quarter of participants (*n* = 245, 24%) could not determine the state of consciousness, while 20% (*n* = 202) failed to identify the absence of respiration. A larger proportion (*n* = 351, 34%) were unable to determine the absence of circulation. Consequently, a large majority (*n* = 833, 82%) could not recognize sudden death, and the same proportion did not know the correct subsequent action to take (Table 3).

**Table 3:** Knowledge, attitudes, and awareness regarding BLS (*n* = 1,021).

Characteristics	**Number of correct responses**	**Number of incorrect responses**
Concerns preventing cardiac massage	817 (80%)	204 (20%)
Signs of sudden cardiac arrest	817 (80%)	204 (20%)
Determining consciousness state	776 (76%)	245 (24%)
Determining absence of respiration	819 (80%)	202 (20%)
Determining absence of circulation	670 (66%)	351 (34%)
Witnessed sudden death	188 (18%)	833 (82%)
Action taken in sudden death	188 (18%)	833 (82%)
Definition of cardiac massage	314 (31%)	707 (69%)
Conduct respiration and cardiac massage	973 (95%)	48 (5%)
Action if family or friend faints	992 (97%)	29 (3%)
Action if stranger faints	986 (97%)	35 (3%)
Knows cardiac massage technique	441 (43%)	580 (57%)
Received BLS training	480 (47%)	541 (53%)
Location of BLS training	527 (52%)	494 (48%)
BLS applications for heart stop	602 (59%)	419 (41%)
Rate of cardiac massage ventilation	278 (27%)	743 (73%)
Area for cardiac massage	412 (40%)	609 (60%)
Rate of cardiac massage	212 (21%)	809 (79%)
Force for heart massage	314 (31%)	707 (69%)
Knowledge of defibrillator	409 (40%)	612 (60%)
Knowledge of AED or pacemaker location	347 (34%)	674 (66%)

Despite most participants (*n* = 973, 95%) correctly conducting resuscitation maneuvers, a significant knowledge gap was evident regarding its theoretical understanding. Over two-thirds (*n* = 707, 69%) did not know the definition of cardiac massage. Furthermore, over half of the participants lacked practical knowledge, with 57% (*n* = 707) unable to demonstrate the cardiac massage technique and 53% (*n* = 541) reporting they had not received any prior BLS training. Nearly half (*n* = 494, 48%) were unable to identify where to obtain training, and 41% (*n* = 419) failed to identify the applications of BLS for cardiac arrest (Table 3).

Knowledge of the correct technical aspects of BLS was notably low. Most participants were unaware of the correct cardiac massage–ventilation ratio (*n* = 747, 73%), proper anatomical area for performing compressions (*n* = 609, 60%), and correct compression rate (*n* = 809, 79%). Furthermore, 69% (*n* = 707) could not identify the required force for effective cardiac massage (Table 3).

Furthermore, knowledge of automated external defibrillators (AEDs) was limited. The majority of participants (*n* = 612, 60%) had no knowledge of defibrillators, and 66% (*n* = 674) were unaware of the location of AEDs or pacemakers (Table 3).

### BLS perception and practice

**Figure 1:**
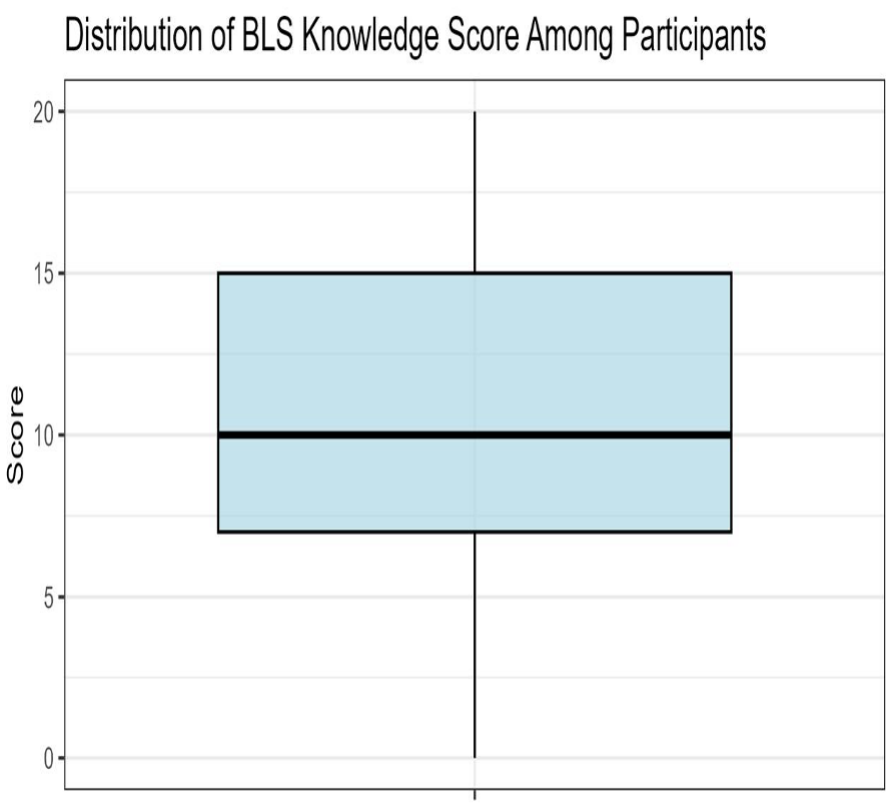
Boxplot of BLS knowledge score.

The participants’ average BLS knowledge score was 10.81 ± 4.94, with a symmetric distribution suggesting approximate normality (Fig. 1). A total of 736 participants (72%) were aware of BLS, with educational institutions being the main source of learning (*n* = 347, 34%). In addition, 912 participants (89%) recognized the importance of BLS, and 884 (87%) considered BLS training to be necessary (Table 4).

**Table 4:** BLS knowledge as perceived by participants (*n* = 1,021).

**Characteristics**	**Frequency (%)**
**Have you ever heard of BLS? BLS awareness**	
*No*	285 (28%)
*Yes*	736 (72%)
**Knowledge source**	
*Educational institution [school or university]*	347 (34%)
*Family and friends*	49 (5%)
*No awareness as such*	285 28%)
*Social media*	200 (20%)
*Television*	29 (3%)
*Workplace*	111 (10%)
**Perceives BLS as important**	
*I don’t know*	90 (9%)
*No*	19 (2%)
*Yes*	912 (89%)
**Believing BLS is necessary**	
*I don’t know*	101 (10%)
*No*	36 (4%)
*Yes*	884 (87%)

**Figure 2:**
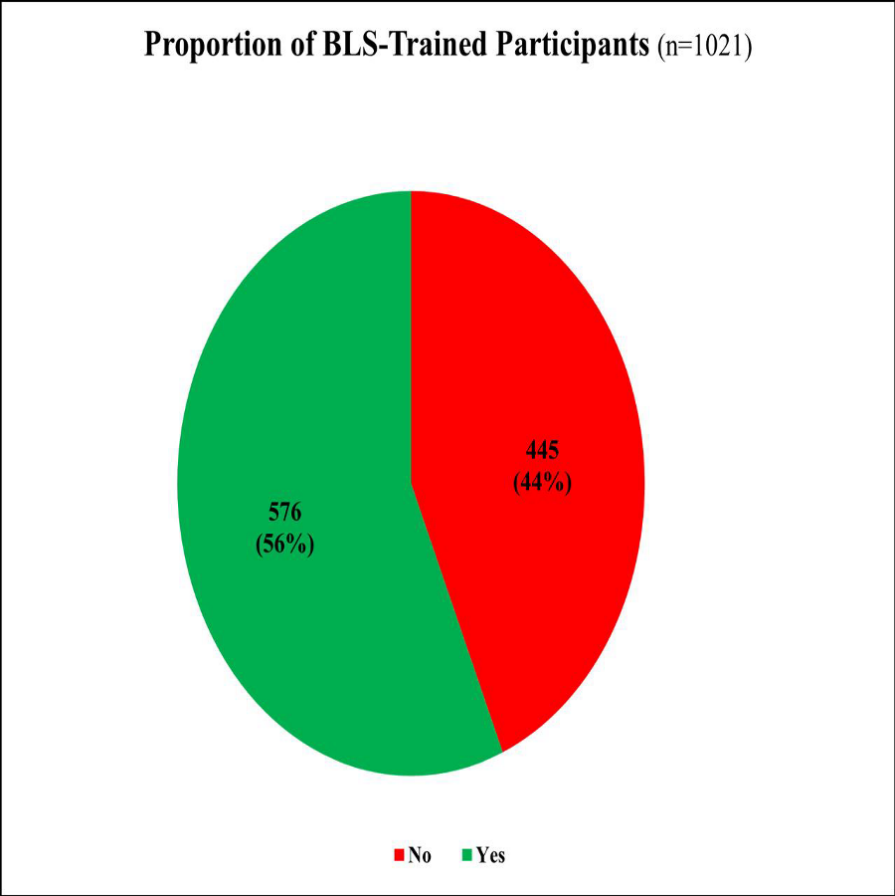
BLS-trained participants.

Half of the participants (*n* = 576; 56%) had received BLS training, with 308 (30%) trained more than two years ago (Fig. 2). Training was optional for 370 (36%) and mandatory for 206 (20%). Regarding training format, only 52 (5%) underwent hands-on practical training. Notably, just 11% of the participants encountered a real-life CPR situation (Table 5).

### Factors influencing BLS knowledge scores

Multiple linear regression analysis revealed several significant predictors of BLS knowledge scores. Socioeconomic factors showed strong associations, with higher income groups demonstrating progressively better scores compared to those earning <5,000 SAR (5,000–9,999 S AR: β = 1.60, *p* < 0.001; 10,000–15,000 SAR: β = 1.58, *p* < 0.001; >15,000 SAR: β = 2.48, *p* < 0.001). Educational qualification also held importance, as bachelor’s degree holders scored higher than those having high school education or less (β = 0.68, *p* = 0.041). Students outperformed unemployed participants by 2.55 points (*p* < 0.001).

Lifestyle factors were also influential: participants engaging in regular moderate-to-intense physical activity (≥5 times/week) scored 0.82 points higher (*p* = 0.019), and current smokers scored 1.01 points higher (*p* = 0.029). Conversely, having children was associated with 2.31-point lower scores (*p* = 0.002) (Table 6).

## Discussion

The skills and knowledge related to BLS are crucial to the public and communities because they are essential for saving lives. The major findings reveled that among 1,021 participants 72% were aware of BLS, 56% had prior training, and only 5% had hands-on practice. Educational institutions and social media were the main knowledge sources. Higher BLS knowledge was positively associated with education, income, and regular physical activity, while parental status showed a negative association. These findings illustrate the urgent need for enhanced public exposure to practical BLS training, since having theoretical knowledge without practical skills can prevent efficient emergency response.

This study showed that 72% of participants had an awareness of BLS, while only 56% had received training. These findings align with regional studies demonstrating growing emergency preparedness awareness, as the study by Sayed et al. (2024) where 85% acknowledged CPR’s life-saving role (Sayed et al., 2024). However, the observed awareness level falls below Saudi Arabia’s national average (>90%) (Alghamdi et al., 2024), yet it exceeds the concerning 49% competency rate among Jazan University students (Ahmad et al., 2018). This regional disparity may reflect healthcare accessibility challenges, as 68% of Jazan residents reside in medically underserved areas (Almalki & Ashdown, 2024). A cross-sectional study from India (*n* = 520) reported that 61.9% identified a lack of awareness regarding BLS, while 92.1% emphasized that BLS training should be incorporated into the medical curriculum (Aroor et al., 2014). These findings suggested the need of incorporating BLS modules into school and university curricula. Structured hands-on training sessions, regular refresher courses, and simulation-based workshops should be implemented to strengthen practical BLS skills and ensure long-term retention among the population.

The development of practical BLS skills with positive attitudes appears more crucial than theoretical knowledge alone. While 56% of participants reported prior BLS training, only 5% had received exclusively hands-on instruction. This highlights a clear imbalance, where the majority lacked sufficient opportunities to practice skills in real-life scenarios. Such a practical training deficit aligns with the study by Almutairi et al. (2023) who found that even trained individuals frequently demonstrated poor CPR execution due to lack of practical skills. Similarly, Alsabri et al. (2024) reported that 70% of participants feared committing errors during CPR despite theoretical familiarity, underscoring the inadequacy of knowledge-only approaches instead of hand-on training (Tadesse et al., 2022). In Jazan’s context, although our study revealed a higher training rate compared to Al-Turki et al. (2008) (56% *vs.* 20%), likely reflecting recent public health initiatives, predominance of non-practical training formats suggests that these programs should shift toward competency-based, hands-on skill development to bridge the gap between knowledge and effective performance (Jarrah, Judeh & AbuRuz, 2018).

Our analysis identifies educational institutions (34%) and social media (20%) as the predominant sources of BLS knowledge, consistent with findings from Alrasheedi et al. (2022). This pattern becomes particularly concerning when examining AI-Turki et al.’s study of King Saud University students, where television (24%) and movies (21%) served as primary CPR learning resources, while only 12.1% had participated in formal training programs (Al-Turki et al., 2008). Such heavy reliance on informal, non-standardized sources highlights an urgent need for systemic integration of competency-based BLS curricula across educational institutions and community programs. The current knowledge acquisition paradigm—where entertainment media and unverified digital platforms supplement (and often substitute for) structured training—demonstrably fails to ensure procedural competency, as evidenced by persistent skill gaps even among trained individuals (Almutairi et al., 2023). To improve access to trusted sources of BLS knowledge and strengthen attitudes and practices, it is essential to shift from informal learning platforms toward structured, evidence-based training. Spaced e-learning, which delivers content in shorter, repeated intervals, has been shown to enhance long-term knowledge retention more effectively than massed learning approaches (Ranjbar et al., 2024).

**Table 5:** Characteristics of BLS training among participants (*n* = 1,021).

**Characteristics**	**Frequency (%)**
**BLS trained**	
*No*	445 (44%)
*Yes*	576 (56%)
**Time of BLS training**	
*Yes, less than two years ago*	268 (26%)
*Yes, more than two years ago*	308 (30%)
**Learning mandatory or optional**	
*I did not learn*	445(44%)
*Mandatory*	206 (20%)
*Optional*	370 (36%)
**Training method**	
*Practical*	52 (5%)
*Theoretical*	131 (13%)
*I did not learn*	445 (44%)
*Both*	393 (38%)
**Faced CPR situation**	
*No*	908 (89%)
*Yes*	113 (11%)

**Table 6:** Determinants of BLS knowledge score among the participants.

	**Knowledge score**		
Predictors	**Beta (β)**	**95% CI**	***P***
**Age**	−0.01	−0.06–0.03	0.612
**Sex (reference: female)**			
*Male*	0.02	−0.78–0.82	0.962
**Weight**	0.00	−0.01–0.02	0.580
**Height**	0.03	−0.01–0.06	0.155
**Nationality (reference: non-Saudi)**			
*Saudi*	0.40	−1.44–2.23	0.673
**Residence (reference: rural)**			
*Urban*	−0.05	−0.61–0.52	0.876
**Marital status (reference: single)**			
*Married*	0.56	−1.11–2.22	0.513
*Divorced/widow*	1.03	−1.54–3.60	0.432
**Income (reference: less than 5,000 SR)**			
*Between 5,000–9,999 SR*	1.60	0.75–2.44	**<0.001**
*Between 10,000 –15,000 SR*	1.58	0.70–2.46	**<0.001**
*>15,000 SR*	2.48	1.69–3.26	**<0.001**
**Education (reference: high school or lower)**			
*Bachelor*	0.68	0.03–1.33	**0.041**
*Postgraduate*	0.79	−0.69–2.26	0.297
**Occupation (reference: unemployed)**			
*Employee*	0.82	−0.10–1.73	0.082
*Student*	2.55	1.60–3.49	**<0.001**
**Risky sports (reference: no)**			
*Yes*	0.36	−0.35–1.08	0.322
**Physical activity (reference: no physical activity)**			
*Moderate to intense physical activity (*≤*30 min, 5x/ week)*	0.82	0.14–1.51	**0.019**
*Moderate to intense physical activity (<30 min, 5x/ week)*	0.27	−0.53–1.07	0.504
**Smoking status (reference: never)**			
*Ex-smoker*	−0.04	−1.21–1.14	0.951
*Current*	1.01	0.10–1.91	**0.029**
**Children (reference: no)**			
*Unmarried*	−0.03	−1.17–1.11	0.963
*Yes*	−2.31	−3.79–−0.83	**0.002**
**Chronic diseases (reference: no)**			
*Yes*	0.20	−0.64–1.05	0.636
**Family chronic diseases (reference: no)**			
*Yes*	0.33	−0.28–0.94	0.286

**Notes.**

Bold formatting is used to visually guide readers toward statistically significant findings (*p* < 0.05).

Our study identified substantial socioeconomic disparities in BLS knowledge, with the highest income bracket (>15,000 SAR) demonstrating a 2.48-point knowledge advantage over the lowest income group (<5,000 SAR; *p* < 0.001). This finding reinforces Alshammari et al.’s (2022) work on socioeconomic determinants of health knowledge accessibility (Almalki & Ashdown, 2024) and is corroborated by Subki et al. (2018) who found that income significantly predicted BLS test performance (*p* = 0.04). The global nature of this disparity is evidenced by Weiner et al. (2010) Boston-based study, where higher-income individuals (>$40,000 annually) showed 2.15 times greater odds (95% CI [1.17–3.95]) of CPR training completion.

Our findings demonstrate a strong positive association between educational qualification, income level, and BLS knowledge. Bachelor’s degree holders had significantly higher score than participants, having high school education (β = 0.68, *p* = 0.041), mirroring patterns observed in Weiner et al.’s study where college graduate patients showed more knowledge about BLS. Graduated patients showed 2.70–fold greater odds (95% CI [1.52–4.78], *p* = 0.0005) of AED recognition and handling (Ranjbar et al., 2024). This association is further supported by Ng et al. (2023) findings of significantly higher CPR training prevalence among more educated individuals (*p* < 0.001). The educational advantage was particularly pronounced among students who scored 2.55 points higher than unemployed participants (*p* < 0.001) (Ng et al., 2023). These results align with those of Srinivasan et al. (2021) who documented improved BLS understanding among students receiving curriculum-based instruction. This suggests that academic progression enhances both theoretical knowledge and practical preparedness for life-saving procedures. Additionally, students displayed a positive attitude and strong willingness to undergo CPR training, emphasizing the value of integrating such programs into educational settings. These results suggest a dual mechanism: education enhances both cognitive understanding of life-saving skills and health literacy, while higher income facilitates access to training opportunities, creating a compounding advantage for socio-economically privileged groups.

The finding that participants engaging in moderate-to-intense physical activity (≥5 times/week) scored 0.82 points higher in BLS knowledge (*p* = 0.019) may be attributed to multiple factors: (1) Health consciousness as physically active individuals are more likely to seek first aid training (OR = 1.42, 95% CI [1.15–1.76]) (Rahmati et al., 2025); (2) Cognitive benefits—since aerobic exercise enhances memory and procedural learning by 12–15% (Prathap, Nagel & Herting, 2021) (3) Socioeconomic links—as fitness engagement often correlates with higher disposable income and access to training (Puciato & Rozpara, 2021); and (4) Psychological readiness, provided that regular exercisers exhibit 30% greater physical self-efficacy (Qiu et al., 2025). While residual confounding (*e.g.*, education/income) cannot be ruled out, these findings suggest that integrating BLS training into wellness programs (*e.g.*, gyms, workplaces) could leverage this association to improve community preparedness.

Our study revealed two particularly novel and intriguing findings. First, we observed a significant negative association between having children and BLS knowledge scores (−2.31, *p* = 0.002), suggesting that parental responsibilities may hinder participation in training. This underscores the need for family-friendly BLS programs—flexible, accessible training options that accommodate parents’ schedules while fostering life-saving skills at home and in community settings. Second, contrary to expectations, current smokers scored 1.01 points higher (*p* = 0.029) on BLS knowledge assessments. This surprising result may reflect smokers’ heightened awareness of health risks, possibly due to increased exposure to health messaging or personal concerns about their habits (Zhang et al., 2024). Given the unexpected nature of this association, future behavioral research should investigate the underlying mechanisms—such as health literacy patterns or motivation for emergency preparedness—among smokers.

In our study, although majority knows about cardiac arrest but knowledge of key BLS terms and equipment was limited, as 69% of participants did not know the definition of cardiac massage and 60% were unfamiliar with defibrillators. This is consistent with the findings of Almesned et al. (2014) who emphasized the importance of familiarity with abbreviations to save time during emergencies. Their study reported that 20% of responders did not know that BLS stood for “basic life support”; 66% were unaware that AED meant “automated external defibrillator”; and only 59% correctly identified EMS as “Emergency Medical Service”. Such deficiencies highlight the critical gap in understanding essential terminology and the use of life-saving devices, which may hinder timely and effective intervention in cases of sudden cardiac arrest.

In our findings, over half of the participants lacked practical knowledge of BLS: more than half did not know the cardiac massage technique, correct cardiac massage–ventilation ratio and had not received BLS training. These results are in line with findings of a recent research that over half (53.3%) had no prior CPR training, and 55.6% had never witnessed CPR being performed. These results showed that most participants exhibited either poor or average levels of knowledge, with critical deficiencies in CPR skills mainly due to lack of education and awareness (Alatawi, 2025). Amro et al. (2024) also reported that only 56% (n = 130) of participants with training correctly identified the recommended 30:2 compression-to-ventilation ratio, highlighting the role of CPR training in effective knowledge of cardiac arrest management.

### Strengths and limitations

This study has several notable strengths. This study benefits from a large, diverse sample across urban and rural areas in Jazan, offering valuable regional insight into public awareness for emergency interventions. For instance, our findings fall below the national average in Saudi Arabia (>90%) but remain higher than the 49% competency rate reported among Jazan University students, underscoring regional gaps even within the same country. The application of a standardized questionnaire and rigorous statistical techniques, such as multiple linear regression, improves the reliability of the results and facilitates the identification of significant sociodemographic determinants of BLS knowledge.

Meanwhile, the study has certain limitations. First, the study is confined to Jazan region only, thus the research may not accurately reflect the entire population of Saudi Arabia. The cross-sectional study design collected data at one specific moment and through online plateform, hence limiting the capacity to determine causal correlations among variables and the findings may not be fully generalizable to the wider population. The study did not differentiate whether the number of children (*e.g.*, one *versus* more than one) is a potential risk factor for BLS, but instead only considered the presence or absence of children. This limits the depth of interpretation and may have influenced the associations observed. Moreover, knowledge scores were dichotomized using a 50% cutoff, whereby participants scoring ≥50% were classified as knowledgeable and those scoring <50% as not knowledgeable. This binary categorization was adopted instead of stratifying into multiple levels (poor, moderate, good) to ensure clarity and simplicity in interpretation. Furthermore, dependence on self-reported questionnaires may result in response bias. A significant weakness is the lack of an objective evaluation of practical BLS abilities; hence, the study cannot ascertain whether individuals with prior training have the capability to administer CPR effectively in real-life situations. The questionnaire included items asking whether respondents believe BLS is important and necessary; it did not specifically explore broader perceptions such as confidence in performing BLS, perceived barriers, or attitudes toward its practical implementation.

Future research should consider longitudinal study designs to assess changes in BLS knowledge, skills, and attitudes over time and to explore causal relationships. Further studies could also investigate barriers to access hands-on BLS training, particularly in underserved or rural areas. The studies should also evaluate the effectiveness of different training delivery methods, including digital platforms, public campaigns, and school-based programs.

## Conclusion

Socioeconomic factors such as income, education, smoking status, academic background, and physical activity were positively associated with BLS knowledge, while having children showed a negative association. These findings illustrate the urgent need for hands-on sessions and broader public education initiatives targeted for vulnerable and underserved populations, including parents, the unemployed and low-income individuals to improve cardiac emergency outcomes in Saudi Arabia.

## Supplemental Information

10.7717/peerj.20678/supp-1 Supplemental Information 1 Survey Questionnaire (English)

10.7717/peerj.20678/supp-2 Supplemental Information 2 Raw data All birds which did not show normal growth in the first examination, but did show normal growth in the second. These birds were used for statistical analysis to compare colony A and colony B.

## Additional Information and Declarations

### Clinical Trial Registration

The following information was supplied regarding Clinical Trial registration: REC-46/04/1203.

### Data Availability

The following information was supplied regarding data availability: The raw measurements are available in the Supplementary File.
