# Supplementary material for: Socioeconomic disparities in basic life support awareness and training among Saudi adults: a cross-sectional study
Source: PeerJ. 2026 Jan 27;14:e20678. doi: 10.7717/peerj.20678 (PMC12857555; doi:10.7717/peerj.20678)
Supplement: Supplemental Information 1 [file peerj-14-20678-s001.pdf]

Section 1: Agreement for participating

.1- **Do you agree to participate in this Survey?**

Yes

No

Section 2: Demographic data

1. Age
(e.g., 22)

2. Weight (kg)
(e.g., 92)

3. Height (cm)
(e.g., 177)

4. Gender

- Male
- Female

5. Education level

- High school or less
- Bachelor's degree
- Postgraduate

6. Employment status

- Employed
- Unemployed
- Student

7. Family monthly income

- Less than 5,000
- 5,000 – 9,999
- 10,000 – 15,000
- More than 15,000

8. Marital status

- Single

- Married
- Divorced
- Widowed

9. If married, do you have children?

- Yes
- No
- Not married

10. Occupation sector

- Military
- Healthcare
- Agricultural
- Educational
- Tourism
- Industrial
- Financial services
- Self-employed / Free trade
- Legal
- Student
- Unemployed

11. Place of residence

- Village
- City

12. Nationality

- Saudi
- Non-Saudi

13. Have you ever encountered a case requiring CPR?

- Yes
- No

14. Do you have chronic health problems (such as hypertension, heart disease, morbid obesity, or diabetes)?

- Yes
- No

15. Do any of your family members have chronic health problems (such as hypertension, heart disease, morbid obesity, or diabetes)?

- Yes
- No

16. Are you a smoker?
(Smoking includes cigarettes, shisha, hookah, electronic shisha/vape, and pipe)

- Yes
- No
- Former smoker

17. Physical activity level

- No physical activity
- Moderate to vigorous physical activity for at least 30 minutes, 5 times a week
- Moderate to vigorous physical activity for less than 30 minutes, 5 times a week

18. Do you practice any of the following sports (or similar)?
(Weightlifting – Boxing – Surfing – Diving – Skydiving – Football – Horse riding – Swimming)

- Yes
- No

Section 3: Level of Knowledge and Practice Regarding Basic Life Support (BLS) Skills

1. **Have you ever heard about the Basic Life Support (BLS) course?**
   Yes
   No
2. **If you answered "Yes" to the previous question, where did you hear about it?**
   Social media
   Television
   Family and friends
   Educational institution (school or university)
   Workplace
   No, I have not heard about it
3. **Do you think learning Basic Life Support (BLS) skills is important?**
   Yes
   No
   I don't know

4. **Do you think there is a need in your surroundings for learning Basic Life Support (BLS) skills?**
   Yes
   No
   I don't know

5. **Have you ever learned or trained in Basic Life Support (BLS) skills?**
   Yes, more than two years ago
   Yes, less than two years ago
   No

6. **Was your learning compulsory or optional?**
   Compulsory
   Optional
   I did not learn

7. **What was the training method?**
   Theoretical
   Practical
   Both
   I did not learn

Section 4: Questions About Your Knowledge and Perspective Regarding Basic Life Support (BLS) Basics

1. **What concerns may prevent you from giving cardiac massage to your friends, relatives, or a stranger?**

- Making a mistake
- Causing bone fractures
- Causing harm to internal organs
- Stopping a working heart
- Punishment due to legal reasons
- Contamination by blood or vomit
- Contracting a contagious disease

2. **Which of the following may be a sign of sudden cardiac arrest?**

- Loss of consciousness

- Discontinuation of breathing
- Discontinuation of circulation (no heartbeat and no pulse)
- Cyanosis
- Nausea
- Chest pain
- Weakness of the body
- The individual is not moving
- I do not know

3. **How can the level of consciousness of an individual be determined?**

- No response when called
- No response when touched
- Not moving at all
- I do not know

4. **How can the absence of breathing be determined?**

- No respiratory movement
- No breathing sound
- No air coming out of the mouth
- No mist appears on a mirror placed in front of the mouth
- I do not know

5. **How can the absence of circulation be determined?**

- Signs of poor circulation
- No pulse felt in the neck vessels
- No pulse felt in the arm vessels
- I do not know

6. **Have you ever witnessed a sudden death? If yes, who was the person?**

- A family member
- A friend or acquaintance
- A stranger
- I have not witnessed a sudden death before

7. **If you have witnessed a sudden death, what did you do? (If not, choose "I have not witnessed a sudden death before")**

- I began to give cardiac massage
- I performed mouth-to-mouth ventilation
- I performed both cardiac massage and mouth-to-mouth ventilation (CPR)

- I called emergency services (997)
- I asked others to call for help
- I called for help by phone
- I just watched and left
- I have not witnessed a sudden death before

8. **What do you think the correct meaning of “cardiac massage” is?**

- To scrub the chest at certain intervals
- To apply strong compression to the chest at certain intervals
- To scrub the heart directly after opening the chest
- To apply compression directly to the heart after opening the chest
- I have no idea

9. **If sudden cardiac arrest occurs, for whom would you perform breathing and cardiac massage?**

- A family member
- A friend
- A neighbor
- A youth in the gym
- A stranger in a supermarket
- A person with poor hygiene at a bus stop
- A drug addict (glue, hashish, heroin)

10. **If one of your family members or friends lost consciousness, what would you do?**

- I would begin cardiac massage
- I would call emergency services (997)
- I would call someone I know or ask for help
- I would just watch and leave

11. **What would you do if you saw a stranger lose consciousness?**

- I would begin cardiac massage
- I would call emergency services (997)
- I would call someone or ask for help
- I would just watch and leave

12. **Do you know how to give cardiac massage in the case of cardiac arrest and respiratory standstill?**

- Yes

- No

13. **Have you received any training in Basic Life Support (BLS)?**

- Yes
- No

14. **If yes, where did you receive the training?**

- At school
- At university
- During military service
- Driving school
- Resuscitation Society course
- Training from the Ministry of Health
- Municipality training course
- Sports club
- Workplace training
- Media, television, or internet
- Other
- I have not received any training

15. **If you face a person whose heart has stopped, which of the following Basic Life Support methods would you use?**

- Open the airway
- Control breathing
- Perform mouth-to-mouth ventilation
- Perform cardiac massage
- Perform both mouth-to-mouth ventilation and cardiac massage
- I do not know

16. **What is the correct ratio of cardiac compressions to breaths during CPR?**

- 5/1
- 15/2
- 30/2
- I do not know

17. **Which part of the chest should cardiac massage be applied to?**

- Upper part of the chest
- Middle part of the chest
- Lower part of the chest

- I do not know

18. **What is the correct rate of cardiac massage?**

- At least 150 times per minute
- At least 100 times per minute
- At least 50 times per minute
- I do not know

19. **How much force should be applied during cardiac massage?**

- Light pressure, enough to move the rib cage down 1–2 cm
- Moderate pressure, enough to move the rib cage down 5–6 cm
- Strong pressure, enough to move the rib cage down 6–10 cm
- As much force as possible
- I do not know

20. **What do you know about the Automated External Defibrillator (AED) and its use in emergencies for cardiac resuscitation?**

- I have never heard of it before
- I have heard of it before but have not seen it
- It is a device to support breathing
- It is a device to restart the heart after it has stopped

21. **Do you know where an Automated External Defibrillator (AED) or "Pacemaker" can usually be found?**

- Yes
- No
